# MIHIC: a multiplex IHC histopathological image classification dataset for lung cancer immune microenvironment quantification

**DOI:** 10.3389/fimmu.2024.1334348

**Published:** 2024-02-02

**Authors:** Ranran Wang, Yusong Qiu, Tong Wang, Mingkang Wang, Shan Jin, Fengyu Cong, Yong Zhang, Hongming Xu

**Affiliations:** ^1^ Affiliated Cancer Hospital, Dalian University of Technology, Dalian, China; ^2^ School of Biomedical Engineering, Faculty of Medicine, Dalian University of Technology, Dalian, China; ^3^ Department of Pathology, Liaoning Cancer Hospital and Institute, Shenyang, China; ^4^ Key Laboratory of Integrated Circuit and Biomedical Electronic System, Dalian University of Technology, Dalian, Liaoning, China; ^5^ Faculty of Information Technology, University of Jyvaskyla, Jyvaskyla, Finland

**Keywords:** lung cancer, immunohistochemical image, database, image classification, transformer models

## Abstract

**Background:**

Immunohistochemistry (IHC) is a widely used laboratory technique for cancer diagnosis, which selectively binds specific antibodies to target proteins in tissue samples and then makes the bound proteins visible through chemical staining. Deep learning approaches have the potential to be employed in quantifying tumor immune micro-environment (TIME) in digitized IHC histological slides. However, it lacks of publicly available IHC datasets explicitly collected for the in-depth TIME analysis.

**Method:**

In this paper, a notable Multiplex IHC Histopathological Image Classification (MIHIC) dataset is created based on manual annotations by pathologists, which is publicly available for exploring deep learning models to quantify variables associated with the TIME in lung cancer. The MIHIC dataset comprises of totally 309,698 multiplex IHC stained histological image patches, encompassing seven distinct tissue types: Alveoli, Immune cells, Necrosis, Stroma, Tumor, Other and Background. By using the MIHIC dataset, we conduct a series of experiments that utilize both convolutional neural networks (CNNs) and transformer models to benchmark IHC stained histological image classifications. We finally quantify lung cancer immune microenvironment variables by using the top-performing model on tissue microarray (TMA) cores, which are subsequently used to predict patients’ survival outcomes.

**Result:**

Experiments show that transformer models tend to provide slightly better performances than CNN models in histological image classifications, although both types of models provide the highest accuracy of 0.811 on the testing dataset in MIHIC. The automatically quantified TIME variables, which reflect proportions of immune cells over stroma and tumor over tissue core, show prognostic value for overall survival of lung cancer patients.

**Conclusion:**

To the best of our knowledge, MIHIC is the first publicly available lung cancer IHC histopathological dataset that includes images with 12 different IHC stains, meticulously annotated by multiple pathologists across 7 distinct categories. This dataset holds significant potential for researchers to explore novel techniques for quantifying the TIME and advancing our understanding of the interactions between the immune system and tumors.

## 1 Introduction

According to the recent global statistics (1), lung cancer ranks as the second most common cancer worldwide and continues to be the leading cause of cancer-related deaths. In particular, it is estimated to be responsible for approximately 1.8 million deaths worldwide each year, which accounts for 18% of all cancer-related deaths. Immunohistochemical biomarkers play a crucial role in tumor staging and prognostic analysis for non-small cell lung cancer (NSCLC) (2), as they can offer valuable insights into tumor characteristics, such as cell proliferation, angiogenesis, invasion, and immune response. IHC is a method of demonstrating the distribution and localization of antigens (i.e. proteins) in tissue slides using antibody-antigen interaction, which is a standard tool in clinical diagnostics (3). It allows for specific detection of particular cell types, molecular markers, or disease biomarkers, enabling accurate identification of tumor types, grading, and molecular subtypes (4). Therefore, histological quantification in IHC slides holds paramount importance in unraveling the intricacies of tumor immune microenvironment (TIME) development (5), which helps to predict clinical outcomes of lung cancer patients.

The advent of digital pathology scanners has rendered it feasible to quantitatively analyze information related to the TIME within IHC slides. Accurately quantifying molecular subtypes, disease markers, and other indicators present in IHC slides is crucial in tailoring appropriate treatment strategies for cancer patients. However, due to the tissue heterogeneity, traditional machine learning algorithms often fail to provide satisfactory performance in IHC image analysis. Deep learning models that have demonstrated remarkable results in the computer vision domain are becoming promising solutions for IHC image analysis.

Deep learning models can be broadly categorized into two groups: convolutional neural networks (CNN) and transformer models, both of which have demonstrated superior performance across a multitude of image classification tasks. The first CNN model, termed as the LeNet (6), was proposed in 1998, while the explosion of CNN began with the emergence of AlexNet (7) in 2012. After that, a series of well-known CNN models such as VGG (8), ResNet (9), and EfficientNet (10) have been introduced to dominate image classification tasks in computer vision domain. Traditional CNNs progressively reduce the image size through convolution and pooling operations, extracting features layer by layer. However, this sequential processing may potentially lead to the loss of essential global context information. With the recent popularity of transformer models (11), the self-attention mechanism has become a central focus in deep learning applications. The self-attention module computes the response at a given position as a weighted sum of features across all positions, facilitating the efficient capture of contextual information and dependencies through parallel processing (12, 13). Thus, as opposed to CNN models, one of notable advantages of transformers lies in their capability to capture global context information in image feature embedding. The transformer models excel in learning both global and local information, enabling them to effectively capture long-term dependencies within an image. Nevertheless, deep learning models also come with numerous challenges and limitations, including the requirement for extensive datasets and their limited capacity to deal with extremely complex tasks (14). The availability of a substantial amount of accurately labeled data is of paramount importance for effectively training deep learning models. For instance, recent studies demonstrated that transformer models achieve superior results over state-of-the-art (SOTA) CNNs (15, 16). A pivotal factor contributing to this enhanced performance can be attributed to the utilization of larger model sizes and extensive training datasets in these studies (17).

Categorizing tissue regions is fundamental to quantifying essential information in IHC image analysis. This process enables a more profound understanding of the specific components within the tissue and their respective roles, ultimately leading to improved precision in pathological assessments. Due to the memory constraints of graphics processing unit (GPU), the tissue microarray (TMA) core or whole slide image (WSI) is too large to fit on a GPU all at once. The feasible approach for tissue classification involves dividing the TMA or WSI into small image patches for training the deep learning model. From the perspective of TIME expression, the micro information such as cellular distribution or tissue composition is generally easier to be captured by models at the patch level than at the WSI level. Thus, training a classifier specifically on image patches is expected to yield superior or comparable performance compared to training a classifier at the WSI level (18). To be more specific, the large-scale WSI or TMA core is first divided into a large mount of image tiles for tile-level subtyping. Tile-level predictions are then stitched together to form the WSI-level results for quantitative TIME analysis. However, there is a scarcity of publicly available multiplex IHC datasets used for lung cancer TIME quantification. An extensive and comprehensive dataset is highly desired to facilitate the quantitative analysis of IHC images. The main contributions of this study are as follows: (1) We build a large Multiplex IHC Histopathological Image Classification (MIHIC) dataset for lung cancer TIME quantification, which consists of 12 different IHC stained types (i.e., CD3, CD34, CD38, CD20, CD68, CDK4, D2-40, Cyclin-D1, Ki67, FAP, P53, SMA), and 7 annotated tissue categories (i.e., Alveoli, Immune cells, Necrosis, Other, Stroma, Tumor, Background). (2) We benchmark two mainstream deep learning architectures including CNNs and transformer models to evaluate their histological classification performance on our MIHIC dataset. (3) We show that automatically quantified TIME variables have prognostic value for overall survival of lung cancer patients.

The organization of this paper is as follows. Section 2 provides related works about public datasets used for histological image classification. We then detail the MIHIC dataset creation, classification models benchmarking, and TIME quantification and survival prognosis in Section 3. Section 4 provides histological image classification results and survival outcome predictions, followed by discussion in Section 5.

## 2 Related works

Tissue classification has consistently been a pivotal task in histopathological image analysis. Analyzing tissue regions via diversified staining techniques provides various levels of diagnostic insight, offering valuable information for accurate pathological assessments. **Table 1** summarizes existing Hematoxylin and Eosin (H&E), and IHC staining histological image datasets. To identify histological components in colorectal cancer (CRC) slides, Kather et al. (19) built a large tissue classification dataset including NCT-CRC-HE-100K and CRC-VAL-HE-7K, where nine tissue types including adipose tissue, background, debris, lymphocytes, mucus, smooth muscle, normal colon mucosa, cancer-associated stroma, and colorectal adenocarcinoma epithelium were annotated in H&E stained slides. Note that H&E staining, a widely utilized method in paraffin section technology, is used to highlight the presence of cell nuclei and cytoplasmic inclusions in clinical specimens (27, 28). Their dataset includes images with the size of 224×224 pixels (0.5um/pixel) per image, which has been used to train CNN models for tissue classification. Based on this research (19), Zhao et al. (29) quantified tumor-stroma ratio, which is shown as an independent predictor for overall survival in resectable colorectal cancer. The BreAst Cancer Histology (BACH) (21) dataset offered a substantial collection of H&E stained histological images for breast cancer classification, along with a set of WSIs with pixel-wise annotations for breast tumor segmentation. The primary objective of releasing this dataset was to facilitate the classification and precise localization of clinically relevant histopathological classes in both TMAs and WSIs by leveraging a well-annotated dataset. Brancati et al. (22) released an open-source BReAst Carcinoma Subtyping (BRACS) dataset, which is a large annotated cohort of H&E stained images to advance the automatic characterization of breast lesions. The BRACS dataset contains 547 WSIs and 4539 ROIs extracted from WSIs. All the ROIs are annotated into three different lesion types, including benign, malignant and atypical, which are further subtyped into seven categories. Javed et al. (20) proposed a large-scale histological image dataset for tissue phenotyping, which consists of 280K patches extracted from 20 H&E stained WSIs of different CRC patients. They extracted features reflecting cell-cell interactions and evaluated two classification tasks, including patch-level separation and patient-level separation on the dataset. Hosseini et al. (23) introduced a novel digital pathology dataset termed as the ‘Atlas of Digital Pathology’ (ADP). The dataset consists of 17,668 patch images extracted from 100 slides, each annotated with up to 57 hierarchical Histologic Tissue Types (HTTs). Since it encompasses diverse tissue types from various organs, this dataset provides a comprehensive training resource for supervised multi-label learning of HTTs at the patch level within digitized WSIs. It is worth noting that all of these public datasets consist of H&E stained histopathological images rather than IHC histological images.

**Table 1:** Relevant dataset summary.

Contributors	Dataset name	Stain type	Cancer type	Class number
Kather et al. (19) Kather et al. (19) Javed et al. (20) Grand challenge (21) Brancati et al. (22) Hosseini et al. (23)	NCT-CRC-HE-100K CRC-VAL-HE-7K CRC-TP BACH BRACS ADP	H&E	Colorectal cancer Colorectal cancer Colorectal cancer Breast cancer Breast cancer Multiple organs	9 9 7 4 7 Hierarchical
Xu et al. (24) Sharma et al. (25) HER2 challenge contest (26)	Anonymous Anonymous Anonymous	IHC	Colorectal cancer Gastric carcinoma Breast cancer	9 4 4

As illustrated in the **Table 1**, there are also studies that focus on histological classification in IHC stained images. Xu et al. (24) built a colorectal cancer IHC image classification dataset, where tissues are grouped into nine types: tumor epithelium, tumor stroma, adipose, background, debris, lymphocytes, mucus, smooth muscle, and normal mucosa. The training dataset includes 154.4K image tiles established from 242 CD3 and CD8 slides of 121 patients, while the test dataset includes 22.5K image tiles established from 114 slides of 57 patients. Note that CD3 and CD8 refer to the IHC staining, which highlights T-lymphocytes expressing CD3 or CD8 proteins, offering valuable insights into the immune cell composition of tissues. They trained a CNN model to identify different tissue types in the WSI and then quantified CD3 and CD8 T-cells within stroma regions as the biomarker for survival prognosis. Considering the theoretical significance of HER2 as a key prognostic factor and therapeutic target in gastric cancer (30), Sharma et al. (25) built a specialized IHC image classification dataset using gastric carcinoma slides. Malignancy levels in this dataset are annotated as HER2 positive (comprising grades 2+ and 3+) or negative (comprising grades 0 and 1+) in 11 WSIs. The IHC annotations were mapped to the corresponding H&E images which were used to train a CNN model for cancer classification. HER2 challenge contest (26) provided a histological image dataset for HER2 scoring which was reviewed and scored by at least two pathologists. The contest dataset includes 172 WSIs extracted from 86 cases of invasive breast carcinomas, encompassing both H&E- and HER2-stained slides. The majority of teams participating the challenge contest employed CNN-based approaches to predict HER2 scores from IHC slides, which was then compared with human assessments. These studies analyzed datasets that employed a single IHC staining technique, and hence datasets incorporating three or more IHC staining methods are relatively scarce. In addition, to the best of our knowledge, there are no publicly available IHC datasets specifically focused on NSCLC patients in the field.

## 3 Materials and methods

**Figure 1:**
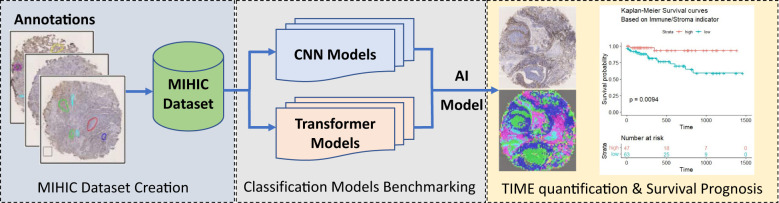
Overview of our study.

**Figure 1** shows the flowchart of this study. As observed in **Figure 1**, we first build a publicly accessible MIHIC dataset based on manual annotations by two pathologists, which includes 309,698 multiplex IHC stained histological image patches. 13 SOTA CNN and transformer models are then evaluated to generate benchmarking results on IHC image classification. Finally, we employ the top-performing model identified in the benchmarking process to quantify TIME variables through entire TMA cores, and explore their associations with survival outcomes of NSCLC patients. The following sections provide details about our study.

### 3.1 MIHIC dataset creation

**Figure 2:**
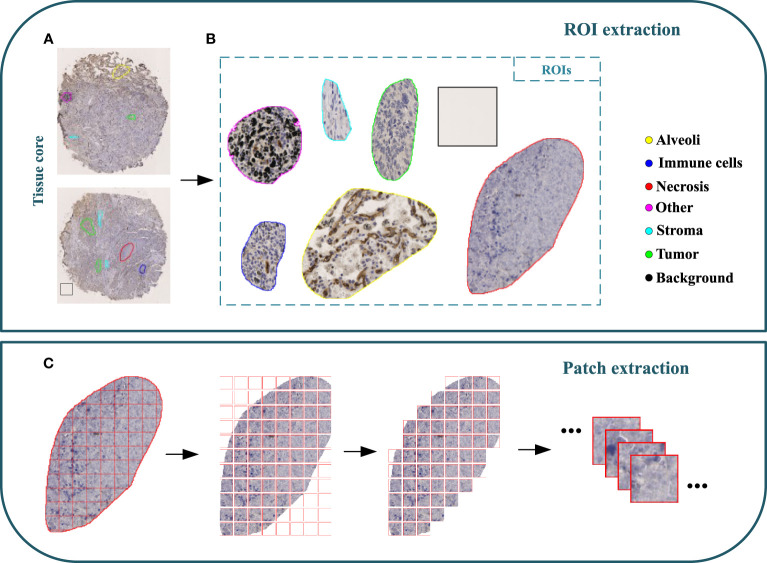
Illustration of MIHIC dataset creation. **(A)** Manually annotated tissue cores, **(B)** different regions of interest (ROIs), **(C)** image patch extraction.

A cohort of 47 TMA sections from 114 patients was collected from Liaoning Cancer Hospital & Institute, where each TMA section has the size of 188,416×110,080 pixels (i.e., 42660.87um×24924.15um) at 40× magnification. TMA sections contain different number of tissue cores, ranging from 28 to 48. Each patient has tissue cores with 12 different IHC stains, including CD3, CD20, CD34, CD38, CD68, CDK4, cyclin-D1, D2-40, Fibroblast Activation Protein (FAP), Ki67, P53, and Smooth Muscle Actin (SMA). Note that CD3 is the marker for T-lymphocytes (31), CD20 is the marker for B-lymphocytes (32), CD34 is the marker for hematopoietic stem cells and endothelial cells (33), CD38 is the marker for plasma cells and certain immune cells (34), CD68 is the marker for macrophages and monocytes (35), CDK4 represents cyclin-dependent kinase 4, involved in cell cycle regulation (36), cyclin-D1 relates the cell cycle progression (37), D2-40 is the marker for lymphatic endothelium (38), FAP expresses in activated fibroblasts (39), Ki67 is the marker for cell proliferation (40), P53 is the tumor suppressor gene product (41), and SMA is the marker for smooth muscle cells and myofibroblasts (42). The selection of these staining slides aims to comprehensively depict the TIME for a more precise diagnosis and prognosis of NSCLC patients. Two pathologists have manually labeled identifiable tissue regions (i.e., without controversy) in TMA sections through visual examination via the Qupath software (43), where six tissue types including Alveoli, Immune cells, Necrosis, Other, Stroma, and Tumor were annotated. Besides the annotated six tissue types, we added one more Background type. **Figure 2A** shows examples of tissue annotations by pathologists, where different tissue regions are enclosed by different color contours.

**Figure 3:**
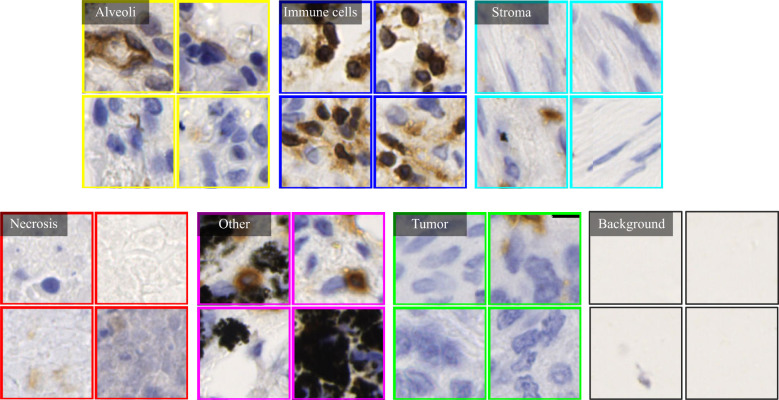
Examples of 7 histological image types included in the MIHIC dataset.

Based on manual annotations by pathologists (see **Figure 2B**), annotated regions of interest (ROIs) corresponding to different tissue types are identified in TMA sections. These ROIs are then divided into a set of non-overlapping image patches (see **Figure 2C**), where each patch has 128×128 pixels. Since some image patches containing a small proportion of annotated tissue regions may negatively influence image classification if they are selected, the MIHIC dataset only includes patches with annotated tissue regions occupying more than 50% of the image patch. In total, 309,698 image patches belonging to 7 different types are generated after tiling all annotated ROIs. **Figure 3** shows examples of 7 histological image patches included in MIHIC dataset. **Table 2** lists the number of different tissue patches generated from TMA cores across 12 different IHC staining methods.

**Table 2:** The number of tissue image patches across 12 IHC staining methods in MIHIC dataset.

Tissues	P53	Ki67	cyclin-D1	CDK4	CD38	CD68	CD34	CD3	SMA	D2-40	CD20	FAP
Alveoli	101	373	189	123	715	514	3179	867	1409	3921	0	398
Immune cells	416	322	549	624	1977	797	455	2926	162	206	1935	471
Necrosis	661	69	874	622	3991	6873	382	5719	757	238	3111	722
Other	7887	4815	5944	4702	3529	3938	6345	4736	4271	4716	4435	6095
Stroma	4012	2885	4866	4839	3163	3214	1871	2377	3182	1959	3439	2170
Tumor	15342	11790	15789	14425	10354	15165	9653	9830	8808	10902	11348	10569

To build histological classification models, we split 309,698 image patches in the MIHIC dataset into three sets: training, validation and testing. Note that image patches extracted from the same annotated tissue region are distributed into the same set, which avoids data leakage during classification model optimization. According to the number of extracted ROIs, training, validation and testing sets accounted for 64%, 16%, and 20%, respectively. **Table 3** lists the number of training, validation and testing image patches across 7 histological image types. It is observed in **Table 3** that tumor image patches occupy the largest proportion among all image types. This is mainly because the majority of TMA cores are tumor regions, which are relatively easier to be identified. By contrast, immune cell image patches occupy the smallest proportion due to their significant expression limited to certain staining methods. Our MIHIC dataset is publicly accessible at https://zenodo.org/records/10065510.

**Table 3:** MIHIC dataset description.

Datasets	Alveoli	Immune cells	Necrosis	Other	Stroma	Tumor	Background
Training	7636	6630	14468	37288	25023	91357	12599
Validation	1262	1817	1857	11188	5362	24625	3149
Testing	2891	2393	7694	12937	7592	27993	3937
Total	11789	10840	24019	61413	37977	143975	19685

### 3.2 Classification models benchmarking

Convolutional neural networks (CNNs) and transformer-based deep learning architectures are SOTA image classification models in computer vision field. In this work, we benchmark a series of SOTA CNN and transformer models for histological classification based on our MIHIC dataset. Since it is difficult to train deep learning models from the scratch due to the high demands of training data scale and hardware resources, we adopt the transfer learning strategy to build histological classification models. The CNN or transformer models initialized with parameters pre-trained on ImageNet (44) are fine-tuned by using our MIHIC dataset, which help to greatly enhance training efficiency and classification accuracy. The following briefly lists 7 CNN models and 6 transformer models which are benchmarked for histological classification on our MIHIC dataset. Note that some of these models have been previously validated, demonstrating strong performance in tissue classifications for various other cancer types (24, 29, 45).

#### 3.2.1 CNN models

- VGG16 (8): is a sequence of successive 3×3 convolutional kernels replacing the larger convolutional kernels in some earlier architectures such as AlexNet (7). This innovation increases the network’s depth while maintaining the same receptive field, ultimately enhancing the model performance.
- GoogleNet (46): is a type of CNN model utilizing Google’s Inception module, which won the 2014 ImageNet competition. Inception modules use multiple filter sizes in parallel to capture information at different scales, allowing the network to learn both fine-grained and coarse-grained features. This model improves the utilization of computing resources inside the network by increasing the depth and width of the network while keeping the computational budget constant.
- ResNet50 (9): is a groundbreaking CNN architecture that was introduced to address the challenge of training very deep neural networks. The key innovation of ResNet model lies in the concept of residual learning. In a residual block, the input to a layer is combined with the output of a previous layer, creating a residual representation that helps to address the problem of vanishing gradients and enable the training of extremely deep networks.
- MobileNetv2 (47): is a lightweight CNN model that has the innovation of utilizing depthwise separable convolutions, where the convolution operation is performed by applying a single filter per input channel (a.k.a., depthwise convolution) followed by using 1×1 filters for cross-channel interactions (a.k.a., pointwise convolutions). This model reduces computational requirements while maintaining the capacity to capture meaningful features.
- HRNet_w18 (48): is able to maintain high-resolution representations through the whole learning process. This model initiates with a high-resolution subnetwork as the initial stage. Subsequently, additional high-to-low resolution subnetworks are incrementally incorporated to establish multiple stages. These multi-resolution subnetworks are then connected in parallel. Unlike ResNet or MobileNet, HRNet combines multi-scale features and retains high-resolution information to improve performance in tasks that require fine-grained details.
- EfficientNet_b0 (10): is an efficient CNN model that uses a novel approach called “compound scaling” to balance the network’s depth, width, and resolution. A specialized building block called “Mobile Inverted Bottleneck (MBConv)” layer is developed to merge depthwise separable convolution with inverted residual blocks. Moreover, this model incorporates the Squeeze-and-Excitation (SE) blocks to further reduce the number of parameters while maintaining or improving performance.
- ConvNeXt_tiny (17): is designed based on group convolutions, which divides the input channels into groups and then applies separate convolutional filters to each group. Grouped convolutions allow the model to capture features with fewer parameters and less computational cost compared to standard convolutional layers, maintaining a balance between model complexity and computational efficiency.

#### 3.2.2 Transformer models

- ViT_base_16 (49): partitions the image into multiple patches, typically the size of 16×16 pixels, and subsequently maps each patch into a fixed-length vector. These fixed-length vectors are then fed into the Transformer encoder for further processing. For image classification tasks, a class token is introduced into the input sequence, and the output associated with this token is used as the final category prediction. ViT has the advantages to capture global dependencies between different image regions in classification tasks.
- Twins-PCPVT_base (50): uses the conditional position encoding introduced in CPVT (51) to replace the absolute positional encoding in PVT. Similar to the CPVT approach, the class token is eliminated, and global average pooling is applied at the final stage to achieve the image-level classification. This amalgamation incorporates the strengths of both PVT and CPVT, resulting in an efficient and straightforward implementation.
- Twins-SVT_base (50): introduces spatially separable self-attention (SSSA) which consists of two components: locally-grouped self-attention (LSA) and global sub-sampled attention (GSA). LSA captures the fine-grained and short-distance information, while GSA deals with the long-distance and global information. Twins-SVT demonstrates the potential of an innovative paradigm, emphasizing that SSSA performs exceptionally well when compared to recent transformer models.
- CrossVit_tiny (52): is a novel dual-branch ViT, which extracts multi-scale feature representations for image classification. It processes small-patch and large-patch tokens with two separate branches of different computational complexity and these tokens are then fused purely by attention multiple times to complement each other. A simple yet effective token fusion module based on cross attention is proposed, which uses a single token for each branch as a query to exchange information with other branches.
- Swin Transformer_tiny (53): is designed to efficiently process and understand images by dividing them into patches and applying hierarchical, multi-head attention mechanisms. It introduces a hierarchical transformer whose representation is computed with shifted windows, which brings greater efficiency by limiting self-attention computation to non-overlapping local windows while also allowing for cross-window connection. Swin Transformer has demonstrated SOTA results in various computer vision tasks.
- CoaT_tiny (54): is capable of acquiring meaningful representations through a modularized architecture. It introduces a co-scale mechanism to image transformer by maintaining encoder branches at separate scales while engaging attention across scales. The specially designed conv-attention module is capable of incorporating relative position information through convolutional operations within the factorized attention module. This results in a substantial improvement in computational efficiency compared to traditional self-attention layers employed in transformers.

#### 3.2.3 Evaluation metrics and settings

To evaluate the patch-level classification performance on the testing set of the MIHIC dataset, we utilized Accuracy (Acc), Recall, Precision (or Positive Predictive Value, PPV), F1-score (27, 55), Negative Predictive Value (NPV), and Area under the Receiver Operating Characteristic (AUC) (56) as evaluation metrics for various deep learning models. Note that Acc is computed as the average accuracy across different classes, whereas recall, precision, and F1-score are computed independently for each class. Acc reflects the overall classification accuracy for all tissue components. Precision signifies the classification accuracy of different tissue components, while F1-score is a comprehensive evaluation metric by balancing recall and precision, offering an overall performance evaluation for each tissue component. NPV quantifies the proportion of actual negative instances that the model correctly predicts as negative. AUC values offer a comprehensive perspective on model performance, aiding in decision-making regarding the trade-offs between the true positive rate and false positive rate. Given the multi-class classification nature of our task, we employ one-vs-rest scheme to compare each class against all others and generate the average ROC curve for each deep learning model. Our experiments were conducted by using the open-source PyTorch library 2.0.1, alongside Python 3.8. When training histological classification models, we implemented various data augmentations including flipping, color jittering, Gaussian blurring, and normalization processes, which enriched the diversity of training data, enhancing the model’s robustness and generalization capabilities. All the models were trained with the SGD optimizer, utilizing the Cosine Annealing technique for dynamic learning rate adjustment.

### 3.3 TIME quantification and survival prognosis

**Figure 4:**
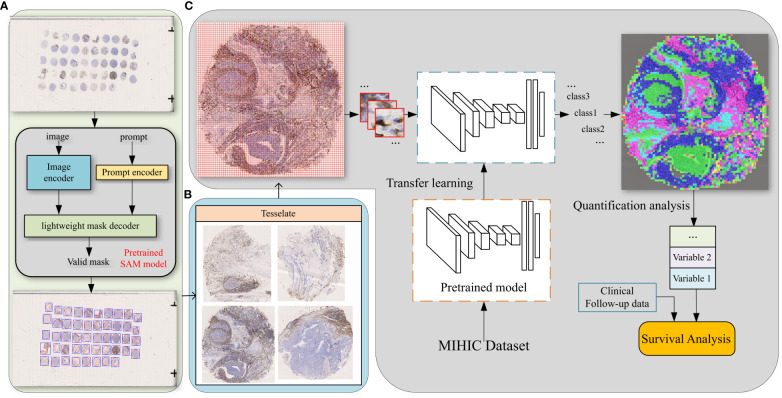
The comprehensive pipeline of quantitative analysis for TMA sections. **(A)** Tissue core extraction, **(B)** extracted tissue core samples, **(C)** diagram of TIME quantification and survival analysis.

**Figure 5:**
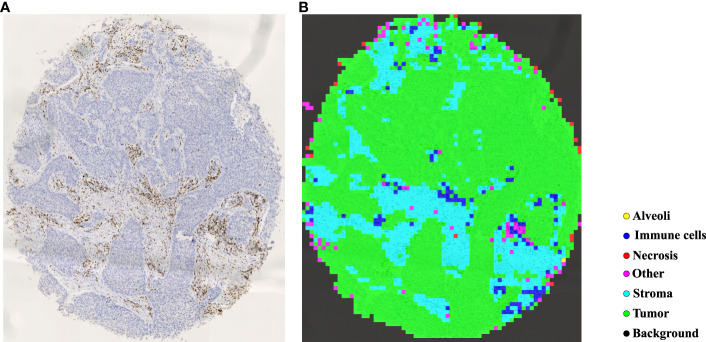
Tissue identification in a tissue core. **(A)** CD3 tissue core, **(B)** tissue classification result.

In TMAs, the spatial locations of tissue cores typically exhibit uncertainty due to the randomness of manual operations involved in creating glass slides. Traditional image processing techniques such as Otsu’s thresholding (57) may lead to inaccuracies and introduce significant noise in extracting tissue cores, which can have detrimental effects on subsequent analyses. To more precisely extract tissue cores in TMAs, we employ a pre-trained image segmentation model, called the segment anything model (SAM) (58), to perform tissue core extraction. Firstly, we downsample the TMA sections by a factor of 64 to obtain the thumbnail images. By applying the SAM, tissue cores within thumbnail images are segmented and stored into a mask. Within the mask, each tissue core region is labeled and indexed sequentially. We then delineate the minimum bounding rectangle for each tissue core region based on its segmented mask. Using the spatial location and scale information obtained from the bounding rectangle, we finally enlarge them by a factor of 64 to obtain the high resolution tissue core image. **Figure 4A** shows the process of tissue core extractions, and **Figure 4B** shows examples of extracted tissue cores. Due to the huge size of the tissue core image, it cannot be directly fed into the classification model. As depicted in **Figure 4C**, the whole tissue core is initially tiled as a set of non-overlapping patches, each consisting of 128×128 pixels. These patches are subsequently normalized through the Z-score normalization before being input into the top-performing tissue classification model (e.g., Swin Transformer_tiny). The tissue classification model is trained via transfer learning on our MIHIC dataset, as detailed in Section 3.2. The patch-level prediction results are stitched together according to their spatial locations in the tissue core, forming the tissue core level classification results. **Figure 5A** shows a CD3 stained tissue core, while **Figure 5B** shows the corresponding tissue classification results using the Swin Transformer_tiny model, where different color pixels indicate different tissue regions.

Based on tissue core level classification, we quantify the proportion of different tissue components on CD3 stained TMA as TIME variables, and then explore their prognostic values to patients’ clinical outcomes. Our quantified TIME variables include: Immune cells/Tumor, Immune cells/Stroma, Immune cells/Necrosis, Tumor/Stroma, Stroma/Tissue core, Immune cells/Tissue core, and Tumor/Tissue core. Note that Tissue-*A*/Tissue-*B* refers to the ratio of tissue *A* (e.g., Immune cells) over tissue *B* (e.g., Tumor) in the TMA core. By considering each of these TIME variables, we employ the correlation function within the R language’s survival package to determine the optimal cutoff value, which divides patients into two groups (i.e., high versus low). Finally, we generate Kaplan-Meier (KM) survival curves for two groups of patients based on the follow-up overall survival information, where the log-rank test is performed to statistically evaluate survival difference.

## 4 Results

In this section, we first provide comparisons of histological classification results on our MIHIC dataset by using different SOTA deep learning models. TIME quantification and survival prognosis for NSCLC patients are then provided.

### 4.1 Classification comparisons

**Figure 6:**
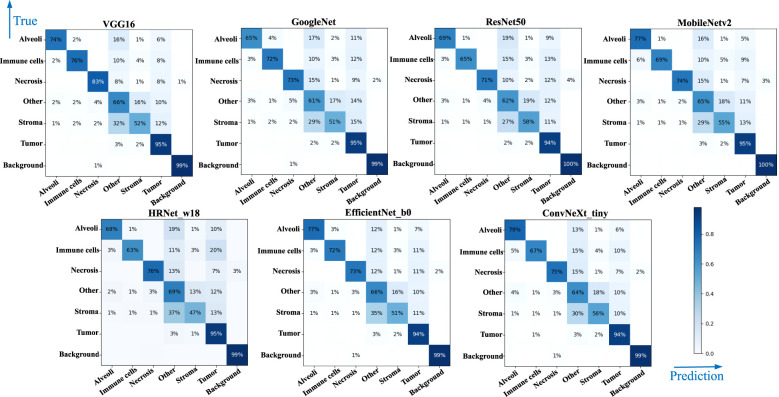
Confusion matrix of different CNN models.

**Table 4** lists comparative classification results by using CNN models. It is observed in **Table 4** that VGG16 provides the highest accuracy of 0.811 among all comparative CNN models, which is 2.7% higher than the lowest accuracy provided by the GoogleNet. VGG tends to provide higher values in terms of recall, precision, and F1-score metrics across different tissue component classifications, although other CNN models may provide the best performance in terms of some evaluation metrics or tissue component classifications. The classification performance of CNN models is influenced by various factors. A more complicated model does not necessarily achieve better results in all tasks due to its higher risk of overfitting (59). The superior performance of VGG16 over other CNN models is likely due to its balanced complexity and generalization on our MIHIC dataset. **Figure 6** shows the corresponding confusion matrix for different CNN models. As depicted in **Figure 6**, all CNN models excel in identifying “Tumor” and “Background” patches, but exhibit significantly lower performance in identifying “Stroma” patches. Distinguishing “Stroma” from “Other” patches poses a challenge, leading to lower performance in these two categories.

**Table 4:** Classification results of CNN models on MIHIC dataset.

	Acc		Alveoli	Immune cells	Necrosis	Other	Stroma	Tumor	Background
VGG16 (8)	**0.811**	Recall	0.739	**0.762**	**0.825**	0.661	0.520	0.948	0.986
		Precision	**0.821**	0.773	0.883	**0.661**	0.598	0.891	**0.978**
		F**l**-score	**0.778**	**0.767**	**0.853**	**0.661**	0.556	**0.919**	**0.982**
		NPV	0.979	**0.996**	0.972	0.901	0.933	**0.968**	1.000
GoogleNet (46)	0.784	Recall	0.648	0.722	0.732	0.610	0.508	0.949	0.988
		Precision	0.783	0.750	0.854	0.624	0.578	0.864	0.950
		F**l**-score	0.709	0.735	0.789	0.617	0.541	0.904	0.969
		NPV	0.984	0.992	0.965	**0.940**	0.923	0.941	1.000
ResNet50 (9)	0.789	Recall	0.691	0.650	0.713	0.620	**0.577**	0.943	**0.996**
		Precision	0.772	0.829	0.887	0.642	0.568	0.872	0.909
		F**l**-score	0.729	0.729	0.791	0.631	**0.573**	0.906	0.951
		NPV	0.992	0.972	**0.987**	0.901	**0.961**	0.908	1.000
MobileNetv2 (47)	0.800	Recall	0.769	0.691	0.736	0.645	0.554	0.946	**0.996**
		Precision	0.775	**0.831**	**0.923**	0.634	0.577	0.888	0.940
		F**l**-score	0.772	0.754	0.819	0.639	0.565	0.916	0.967
		NPV	0.988	0.992	0.983	0.922	0.921	0.936	1.000
HRNet_wl8 (48)	0.798	Recall	0.684	0.630	0.763	**0.686**	0.470	**0.951**	0.991
		Precision	0.808	0.852	0.899	0.620	**0.625**	0.873	0.935
		F**l**-score	0.741	0.724	0.825	0.651	0.536	0.911	0.962
		NPV	0.984	0.988	0.978	0.902	0.912	0.951	1.000
EffcientNet_b0 (10)	0.795	Recall	0.767	0.718	0.730	0.662	0.506	0.939	0.990
		Precision	0.768	0.786	0.916	0.630	0.570	0.882	0.951
		F**l**-score	0.767	0.750	0.813	0.646	0.536	0.910	0.970
		NPV	0.983	0.992	0.951	0.920	0.933	0.961	1.000
ConvNeXt_tiny (17)	0.798	Recall	**0.792**	0.671	0.747	0.644	0.561	0.936	0.986
		Precision	0.737	0.808	0.914	0.623	0.571	**0.898**	0.958
		F**l**-score	0.764	0.733	0.822	0.633	0.566	0.917	0.972
		NPV	**0.996**	0.988	0.970	0.916	0.910	0.966	1.000

The bold values highlight the optimal results under different evaluation criteria.

**Figure 7:**
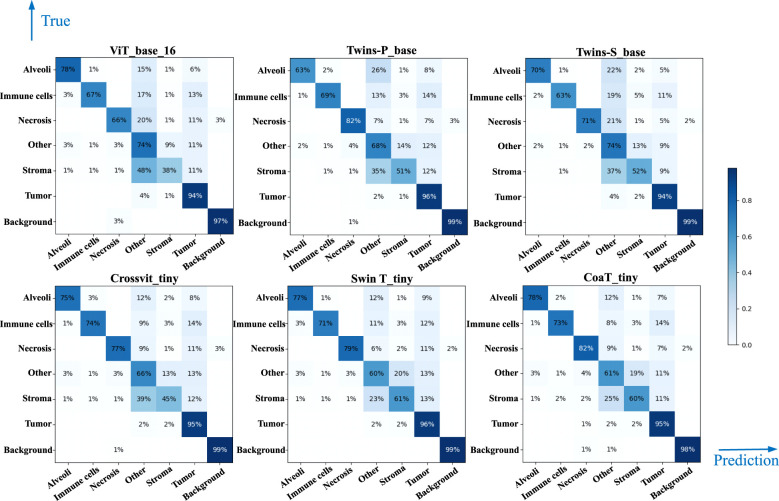
Confusion matrix of different Transformer models.

**Table 5** lists comparative classification results by using transformer models. **Figure 7** shows the corresponding confusion matrix for different transformer models. Similar observations occur in confusion matrices when utilizing transformer models, where “Tumor” patches are effectively recognized, but distinguishing “Stroma” patches remains challenging. It is observed in **Table 5** and **Figure 7** that Swin T_tiny and CoaT_tiny provide the highest classification accuracy of 0.811 among all comparative transformer models, which is 2.9% higher than the lowest accuracy provided by the ViT_base_16. Overall, the Swin T_tiny provides the best performance across different tissue component classifications, although the Twins-S_base also provides comparative performances. The innovative hierarchical design, shifted windows, and efficient use of parameters make the Swin Transformer effective at capturing spatial and contextual information within images, helping it provide a superior performance on our MIHIC dataset.

**Table 5:** Classification results of Transformer models on MIHIC dataset.

	Acc		Alveoli	Immune cells	Necrosis	Other	Stroma	Tumor	Background
ViT_base_16 (49)	0.782	Recall	0.778	0.665	0.656	0.737	0.381	0.936	0.969
		Precision	0.785	0.854	0.878	0.569	**0.656**	0.882	0.938
		F1-score	0.781	0.748	0.751	0.642	0.482	0.908	0.953
		NPV	**0.996**	0.988	0.975	0.905	0.958	0.937	1.000
Twins-P_base (50)	0.810	Recall	0.626	0.689	**0.819**	0.683	0.510	0.956	0.992
		Precision	**0.878**	0.852	0.901	0.641	0.620	0.884	0.944
		F1-score	0.731	0.762	**0.858**	**0.661**	0.559	0.918	0.967
		NPV	0.992	0.980	**0.983**	0.900	0.896	**0.942**	0.996
Twins-S_base (50)	0.805	Recall	0.704	0.633	0.713	**0.739**	0.523	0.940	**0.994**
		Precision	0.855	**0.881**	**0.935**	0.595	0.620	**0.911**	0.959
		F1-score	0.772	0.737	0.809	0.659	0.568	**0.925**	**0.976**
		NPV	0.964	0.988	0.979	**0.915**	0.940	0.928	1.000
Crossvit_tiny (52)	0.798	Recall	0.753	**0.735**	0.767	0.662	0.452	0.951	0.990
		Precision	0.787	0.791	0.900	0.636	0.595	0.872	0.947
		F1-score	0.770	0.762	0.828	0.649	0.514	0.910	0.968
		NPV	0.980	0.988	0.970	0.894	0.937	0.937	1.000
Swin T_tiny (53)	**0.811**	Recall	0.767	0.714	0.787	0.602	**0.609**	**0.961**	**0.994**
		Precision	0.813	0.857	0.922	**0.701**	0.575	0.870	0.954
		F1-score	**0.789**	**0.779**	0.849	0.647	**0.592**	0.913	0.974
		NPV	0.987	**0.996**	0.961	0.895	0.949	0.910	1.000
CoaT_tiny (54)	**0.811**	Recall	**0.779**	0.733	0.816	0.611	0.598	0.950	0.981
		Precision	0.782	0.792	0.881	0.684	0.586	0.889	**0.967**
		F1-score	0.780	0.761	0.847	0.645	**0.592**	0.918	0.974
		NPV	0.992	**0.996**	0.978	0.900	**0.962**	0.923	1.000

Twins-P_base, Twins-S_base and Swin T_tiny represent Twins-PCPVT_base, Twins-SVT_base and Swin Transformer_tiny, respectively.

The bold values highlight the optimal results under different evaluation criteria.

**Figure 8:**
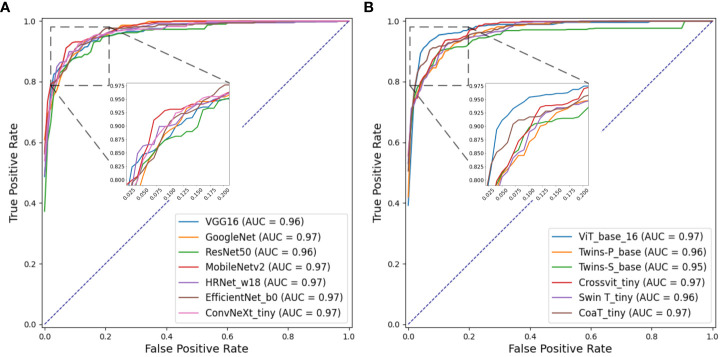
ROC curves of multi-class classification using various deep learning models. **(A)** ROC curves for different CNN models, **(B)** ROC curves for different Transformer models.

**Figures 8A, B** separately show the average ROC curves for various CNN models and transformer models. As shown in **Figure 8**, deep learning models can provide overall good performance in histological tissue classifications, with slight variations in AUC values. By comparing **Tables 4** and **5**, it is found that transformer models generally deliver slightly better performances than CNN models, although both types of models provide the highest accuracy of 0.811. Among all tissue components, the “Stroma” exhibits the least favorable classification result, with the highest precision being 0.656 provided by ViT_base_16. This is mainly attributed to the limited volume of data available under the “Stroma” category. Furthermore, it is found that some “Stroma” patches exhibit features similar to those of the “Other” category, and hence “Stroma” and “Other” are likely to be misclassified (see **Figures 6**, **7**). Although the “Immune cells” constitutes the smallest proportion in the MIHIC dataset, its classification performance is reasonably good across different models. There are two reasons for this. Firstly, immune cells exhibit distinct cellular features compared to other classes. For instance, most of immune cell nuclei have round or oval shapes and are relatively smaller in size, making them generally easier to differentiate. Secondly, the presence of the four stains (CD3, CD20, CD38, CD68), serving as markers for immune cell subtypes, facilitates a more straightforward differentiation process. This emphasizes the advantage of generating the IHC dataset for comprehensive immune cell analysis. Due to the wide abundance of “Tumor” in our TMA cores, pathologists manually annotated a substantial number of instances belonging to this class (see **Table 3**). As observed in **Tables 4** and **5**, the Recall, Precision, and F1-score for the “Tumor” class range from 0.864 to 0.961 among different models, which are relatively higher compared to metrics for other categories such as Alveoli, Immune cells, Necrosis, and Stroma. This suggests that our trained classification models on the MIHIC dataset deliver superior performance in identifying tumors compared to other tissue categories. All deep learning models provide a high NPV above than 0.89 across various tissue categories, demonstrating the effectiveness of our trained models in accurately identifying true negatives during histological tissue classifications.

### 4.2 TIME analysis and prognosis

**Figure 9:**
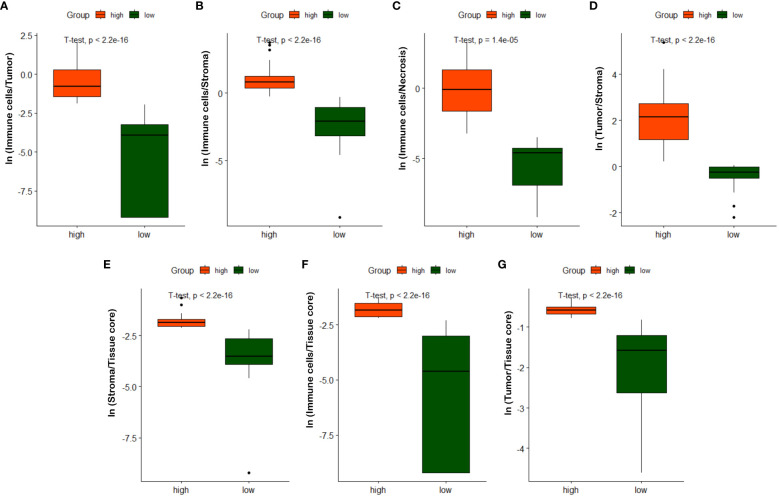
Box plots of quantified TIME variables. **(A)** *ln*(Immune cells/Tumor), **(B)** *ln*(Immune cells/Stroma), **(C)** *ln*(Immune cells/Necrosis), **(D)** *ln*(Tumor/Stroma), **(E)** *ln*(Stroma/Tissue core), **(F)** *ln*(Immune/Tissue core), **(G)** *ln*(Tumor/Tissue core).

As described in Section 3.3, we quantified 7 TIME variables based on tissue core level classification. The patients are then partitioned into two groups (i.e., high versus low) based on the selected cutoff value for each TIME variable. Note that the optimal cut-off for each variable is determined using the maximally selected rank statistic, dividing the patients into two groups with the most significant statistics between each other (29, 60). **Figure 9** shows box plots of quantified variables for patients divided into two groups. Due to the relatively concentrated nature of variable values within the same group, we have applied the natural logarithm to the raw data when creating the box plots, facilitating more clear observations. As observed in **Figure 9**, the high and low groups of patients present statistically significant difference across different TIME variables, highlighting their potentially prognostic values.

**Figure 10:**
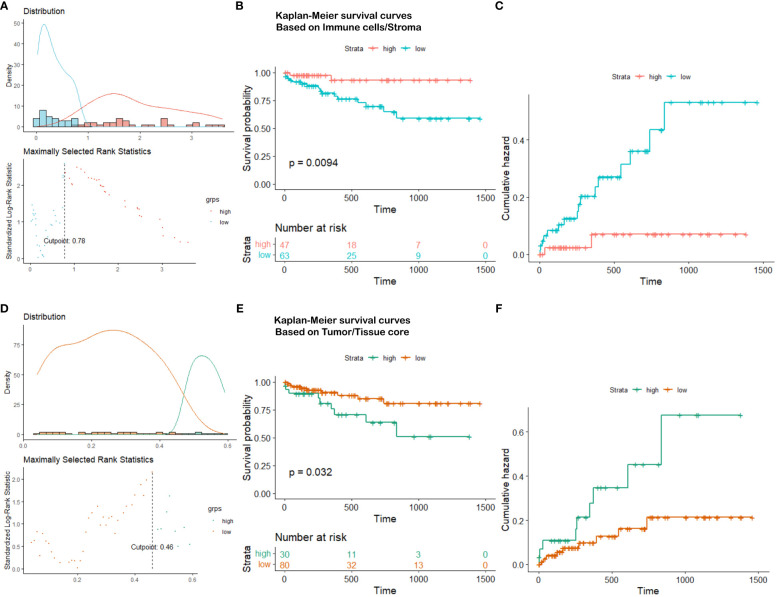
Survival analysis visualization. **(A)** Immune cells/Stroma distribution density and cutoff point selection, **(B)** Kaplan-Meier survival curves based on Immune cells/Stroma, **(C)** Cumulative hazard based on Immune cells/Stroma, **(D)** Tumor/Tissue core distribution density and cutoff point selection, **(E)** Kaplan-Meier survival curves based on Tumor/Tissue core, **(F)** Cumulative hazard based on Tumor/Tissue core.

Based on the partition from TIME variables, we explore survival difference between high and low groups of patients. After excluding patients with missing tissue cores or those with poor image qualities, 110 NSCLC patients with complete clinical information are used for survival prognosis. By generating the KM survival curves and computing log rank test p-values, two TIME variables that are Immune/Stroma and Tumor/Tissue core are found to exhibit significant correlations with overall survivals of NSCLC patients. **Figures 10A** and **D** show the data distribution density and selection of the optimal cutoff values. **Figures 10B** and **E** show the KM survival curves and risk tables based on values of Immune/Stroma and Tumor/Tissue core, respectively. **Figures 10C** and **F** show the corresponding cumulative hazard curves. As shown in **Figure 10B**, the log rank test p-value is 0.0094, which is less than the significance level of 0.05. This suggests that patients with a high Immune cells/Stroma score present a significantly better survival outcomes (i.e., a relatively low risk of death), compared with patients with a low Immune cells/Stroma score. Similarly, as shown in **Figure 10E**, the log rank test p-value is 0.032, which is also less than the significance level of 0.05. This suggests that patients with a high Tumor/Tissue core score present a significantly poor survival outcomes (i.e., a relatively high risk of death), compared to patients with a low Tumor/Tissue core score. In addition, to verify the reliability of this association, we quantify the values of Immune/Stroma and Tumor/Tissue core using all our trained CNN and transformer models. We then utilize them to prognosticate the survival outcomes of NSCLC patients. **Table 6** presents the computed log test p-values of the two variables based on different models. As shown in **Table 6**, all the log rank test p-values are less than or equal to 0.05, indicating the reliable associations between these two variables and overall survival outcomes. Taken together, TIME variables related to the proportions of Immune cells/Stroma and Tumor/Tissue core show potential for predicting survival outcomes for NSCLC patients. These variables offer valuable insights into the interplay between immune cells, tumor components, and the surrounding tissue, which can be indicative of patient prognosis and survival.

**Table 6:** The log-rank test p-values in survival analysis by using two variables quantified based on different deep learning models.

Architectures	Model Names	Immune cells/Stroma	Tumor/Tissuecore
CNN models	VGG16 (8) GoogleNet (46) ResNet50 (9) MobileNetv2 (47) HRNet_w18 (48) EfficientNet_b0 (10) ConvNeXt_tiny (17)	0.004 0.007 0.025 0.005 0.013 0.012 0.022	0.029 0.041 0.029 0.029 0.026 0.049 0.028
Transformer models	ViT_base_16 (49) Twins-P_base (50) Twins-S_base (50) Crossvit_tiny (52) Swin T_tiny (53) CoaT_tiny (54)	0.050 0.022 0.050 0.015 0.009 0.023	0.041 0.032 0.029 0.037 0.032 0.029

## 5 Discussion

In this paper, we built a multiplex IHC Histopathological Image Classification (MIHIC) dataset for lung cancer TIME quantification, which comprises of a total of 309,698 image patches with 12 different IHC stains, meticulously annotated by multiple pathologists across 7 distinct categories. To the best of our knowledge, it is the first publicly available lung cancer histopathological image dataset that incorporates a diverse range of IHC stains. Using the MIHIC dataset, we employed transfer learning techniques and benchmarked 13 SOTA CNN and transformer models for histological image classification. The topperforming model was used to identify different histological components in TMA cores from a cohort of NSCLC patients. The automatic quantification of TMA cores, which aids pathologists in accurately interpreting the TIME, is of great significance for advancing the development of intelligent medicine.

Seven TIME variables were quantified based on tissue core level classification. Two derived TIME variables, namely Immune cells over Stroma and Tumor over Tissue core, exhibit significant correlations with the overall survival of NSCLC patients. The ratio of Immune cells over Stroma demonstrates a significant correlation with the overall survival of NSCLC patients, aligning with prior research. For example, a previous study indicated that tumor-infiltrating lymphocytes (TILs), especially CD8+ T cells, play a crucial role in anti-tumor immune responses, and elevated levels of TILs are linked to a better prognosis in NSCLC (61). Furthermore, immune checkpoint inhibitors, including anti-PD-1 and PD-L1 antibodies, have emerged as a vital element in NSCLC treatment. These medications improve patient survival by reinstating the immune cells’ capability to attack tumors (62). Inflammation within the stroma has been identified as being associated with cancer progression and the survival outcomes of cancer patients. This suggests that chronic inflammation may play a role in the initiation and development of tumors (63). Furthermore, the size of the tumor region has been considered a pivotal factor influencing patient survival, where larger tumors are typically associated with a poorer prognosis (64). This is also consistent with our findings based on the tissue core level classification.

Our study also has certain limitations. The focus of our research is restricted to NSCLC, which limits the generalizability of our constructed MIHIC data cohort to other cancer types. However, quantitative indicators, such as the Immune cells over Stroma score discussed in this paper, have demonstrated significant correlations with prognosis and survival in other cancer types (24). Besides, certain TIME components, like immune cells, are better intricately detected at the cellular level. Patch-level classification for tissue component identification is a relatively coarse approach. This may result in the failure to identify regions where specific immune cells are sparsely distributed. Although our trained deep learning models exhibit statistical significance in overall survival prognosis, there remains significant potential for technical enhancements to accurately quantify categories like immune cells. The intricacy and heterogeneity of the TIME suggests that this field is still in a constant state of evolution. Our present quantitative exploration of the TIME is not exhaustive, highlighting the necessity for more comprehensive quantification of various components and indicators in the future. This serves as a focal point for our ongoing research. Nevertheless, our open sharing of the MIHIC dataset and source code provides significant benefits to research by promoting transparency and reproducibility. More in-depth TIME analysis to explore tumor heterogeneity and its correlations with patients’ treatment responses are expected based on this study.

## Data availability statement

The original contributions presented in the study are included in the article/supplementary material, further inquiries can be directed to the corresponding authors.

## Ethics statement

The studies involving humans were approved by Liaoning Cancer Hospital and Institute. The studies were conducted in accordance with the local legislation and institutional requirements. The ethics committee/institutional review board waived the requirement of written informed consent for participation from the participants or the participants’ legal guardians/next of kin because this is a retrospective study that collects a IHC histological image dataset for lung cancer tumor immune microenvironment quantification.

## Author contributions

RW: Writing – original draft, Software, Methodology, Conceptualization. YQ: Writing – original draft, Resources. TW: Writing – original draft, Software. MW: Writing – original draft, Software. SJ: Writing – original draft, Software. FC: Writing – review & editing, Supervision. YZ: Writing – review & editing, Supervision. HX: Writing – review & editing, Supervision, Project administration, Conceptualization.
